# Human Surfactant Protein A2 Gene Mutations Impair Dimmer/Trimer Assembly Leading to Deficiency in Protein Sialylation and Secretion

**DOI:** 10.1371/journal.pone.0046559

**Published:** 2012-10-03

**Authors:** Yi Song, Guodong Fang, Haitao Shen, Hui Li, Wenbing Yang, Bing Pan, Guowei Huang, Guangyu Lin, Lian Ma, Belinda Willard, Jiang Gu, Lemin Zheng, Yongyu Wang

**Affiliations:** 1 Department of Pathology, Shantou University Medical College, Shantou, Guangdong, People’s Republic of China; 2 Department of Pediatrics, Second Affiliated Hospital of Shantou University Medical College, Shantou, Guangdong, People’s Republic of China; 3 Translational Medicine Center, Second Affiliated Hospital of Shantou University Medical College, Shantou, Guangdong, People’s Republic of China; 4 The Institute of Cardiovascular Sciences and Institute of Systems Biomedicine, School of Basic Medical Sciences, Peking University Health Science Center, Key Laboratory of Molecular Cardiovascular Sciences of Education Ministry, and Key Laboratory of Cardiovascular Molecular Biology and Regulatory Peptides of Health Ministry, Beijing, People’s Republic of China; 5 Proteomics Laboratory, Cleveland Clinic, Cleveland, Ohio, United States of America; University of Saarland Medical School, Germany

## Abstract

Surfactant protein A2 (SP-A2) plays an essential role in surfactant metabolism and lung host defense. SP-A2 mutations in the carbohydrate recognition domain have been related to familial pulmonary fibrosis and can lead to a recombinant protein secretion deficiency in vitro. In this study, we explored the molecular mechanism of protein secretion deficiency and the subsequent biological effects in CHO-K1 cells expressing both wild-type and several different mutant forms of SP-A2. We demonstrate that the SP-A2 G231V and F198S mutants impair the formation of dimmer/trimer SP-A2 which contributes to the protein secretion defect. A deficiency in sialylation, but not N-linked glycosylation, is critical to the observed dimmer/trimer impairment-induced secretion defect. Furthermore, both mutant forms accumulate in the ER and form NP-40-insoluble aggregates. In addition, the soluble mutant SP-A2 could be partially degraded through the proteasome pathway but not the lysosome or autophagy pathway. Intriguingly, 4-phenylbutyrate acid (4-PBA), a chemical chaperone, alleviates aggregate formation and partially rescued the protein secretion of SP-A2 mutants. In conclusion, SP-A2 G231V and F198S mutants impair the dimmer/trimer assembly, which contributes to the protein sialylation and secretion deficiency. The intracellular protein mutants could be partially degraded through the proteasome pathway and also formed aggregates. The treatment of the cells with 4-PBA resulted in reduced aggregation and rescued the secretion of mutant SP-A2.

## Introduction

Human surfactant protein A (SP-A) is an oligomeric glycoprotein synthesized primarily in alveolar type II cells and Clara cells. SP-A belongs to the collectin family which contains four different domains: (1) The N-terminal non-collagenous domain which is involved in intermolecular disulfide bond formation; (2) The collagenous region which is critical for oligomerization, (3) The α-helical coiled coil neck domain which is related to SP-A trimerization, and (4) the carbohydrate recognition domain (CRD) which binds to both lipids and oligosaccharides [1]. SP-A undergoes several post-translational modifications before secretion including N-terminal peptide cleavage, acetylation [2], glycosylation [3], [4], hydroxylation [5], [6] and oligomerization. SP-A monomers can assemble into trimeric subunits which can further form oligomers. The post-translational modifications occur in both the endoplasmic reticulum (ER) and Golgi complex. In the ER, the SP-A protein will be correctly folded, assembled, and modified with the help of molecular chaperones and then transported along the secretory pathway.

SP-A is an important regulator in both surfactant-related functions and innate immunity. It modulates inflammatory processes of the lung [7]. In SP-A gene knockout mice, tubular myelin, an extracellular surfactant structure, can not form properly and these mice are susceptible to various pathogens and environmental insult [8], [9]. In addition, SP-A can bind and aggregate bacterial, fungal, viral, and mycobacterial organisms, directly activate macrophages, enhance in vitro phagocytosis and kill a variety of pulmonary pathogens [10]. In humans, SP-A has two isoforms, SP-A1 and SP-A2 [11], [12]. Although they have very similar structures, many *in vitro* assays indicate that SP-A2 is more biologically active than SP-A1 [13].

Many lung diseases are associated with abnormal SP-A expression in both bronchoalveolar lavage fluid (BAL) and serum, genetic polymorphisms, and gene mutations of SP-A [14]. Recently, two SP-A2 gene mutations (G231V and F198S) have been identified in two large kindred with familial pulmonary fibrosis. These mutations lead to protein instability and ER stress when expressed in vitro [15], [16]. It is unclear how these mutations affect protein processing, metabolism, and cell function. In this study we constructed the wild-type and mutant SP-A2 plasmids to explore the mechanism of protein biosynthesis, secretion, and related biological effects in CHO-K1 cells. We found that SP-A2 mutation impairs protein sialylation and secretion. The accumulated mutant proteins in the cell can partially be degraded via the proteasome pathway and can also form aggregates. This may be due to impaired dimmer/trimer assembly of the mutant proteins. Moreover, 4-PBA, a chemical chaperone, can reduce the aggregate formation and promote mutant protein secretion.

## Materials and Methods

### Materials

Culture medium and fetal bovine serum (FBS) were obtained from HyClone (Thermo Scientific, Rockford, IL). Protease inhibitor cocktail tablets were purchased from Roche Applied Science. Anti-V5 mouse mAb (R960-25) was obtained from Invitrogen, anti-calreticulin (H-170) from Santa Cruz Biotechnology (Santa Cruz, CA); and HRP-conjugated goat anti-mouse and goat anti-rabbit secondary antibodies were obtained from Southern Biotech (Birmingham, AL). Alexa488-goat anti-mouse and Alexa594-goat anti-rabbit antibodies were obtained from Molecular Probes (Invitrogen, Grand Island, NY). All other chemicals and reagents were obtained from Sigma-Aldrich (St Louis, MO) unless otherwise indicated.

### Plasmids Construction and Mutagenesis

For the transient expression in mammalian cells, the pcDNA3.0 vector was used. All PCRs for cloning utilized high-fidelity DNA polymerases and all subclones were confirmed by sequence analysis. Two partial IMAGE cDNA clones, 5184888 and 841707 (Invitrogen) were used to construct a full-length human SP-A2 cDNA. The N-terminal and C-terminal halves were PCR amplified, combined and PCR amplified, cloned into pGEM-T Easy, digested with EcoRI and subcloned into pcDNA3.0. An in-frame V5 epitope tag was constructed after the glutamic acid at amino acid 21 by primer extension mutagenesis and zipper PCR. The DNA sequence of the V5-tag is 5′-GGT AAG CCT ATC CCT AAC CCT CTC CTC GGT CTC GAT TCT ACG-3′. Site directed mutagenesis (QuickChange, Stratagene) was utilized so that the DNA sequence of wild-type SP-A2 clones exactly matched NM_006926.2. SP-A2 neck region (100–133) deletion mutant (SP-A2 d(100–133) ) is constructed by overlap extension PCR. All above primers as described in Table. S1.

### Cell Culture and Transfection

CHO-K1 cells (CCL-61) were purchased from the American Type Culture Collection and maintained in DMEM/F12 with 5% FBS, 100 units of penicillin and 100ug of streptomycin, and incubated at 37°C with 5% CO_2_. Cells were transiently transfected with different expression constructs using FuGENE HD Transfection Reagent (Roche, Basel, Switzerland) according to the manufacturer’s protocol.

### Generation of Stable Cell Lines

Stable CHO-K1 clones were obtained by standard procedures. Briefly, cells with 80% confluence in 6 cm dishes were transfected with the pcDNA3.0 vector or SP-A2 variants plasmids. After selection with 0.5 g/L of neomycin, resistant cells were cloned by limiting-dilution. The expression of V5-tagged SP-A2 variants was confirmed by western blotting.

### SDS-PAGE and Immunoblot Analysis

Protein concentrations in the cell lysates were determined by BCA protein assay (Thermo Scientific, Rockford, IL), according to the manufacturer’s protocol. Protein aliquots were eletrophoresed on 10% SDS-PAGE Bio-Rad minigels and transferred to nitrocellulose (NC) Protran membranes (Whatman, Dassel, Germany). The blots were incubated for 1 hour at RT in blocking buffer (5% dried milk in TBST (150 mM NaCl, 10 mM Tris, pH8.0, 0.1% Tween-20)), and with primary antibody overnight at a dilution of 1∶10,000 (anti-V5 antibody ) in blocking buffer. After washed 4 times in TBST for 5 minutes each, the blots were incubated with secondary antibody at 1∶20,000 in blocking buffer for 1 hour at RT, washed 4 times in TBST for 5 minutes each, and developed with SuperSignal West Pico Chemiluminescent substrate (Thermo Scientific, Rockford, IL) according to the manufacturer’s protocol and exposed with Kodak films.

### NP-40-soluble and NP-40-insoluble Fractionations Assay

CHO-K1 cells in 6-well plates were transiently transfected with V5-tagged SP-A2 constructs (1 µg/well) using the FuGENE HD transfection reagent according the manufacturer’s procedure. At 72 h post-transfection, cells were washed once with 2 ml of ice-cold PBS and harvested in ice-cold lysis buffer (100 mM NaCl, 50 mM HEPES, pH 7.4, 1.5 mM MgCl_2_, 0.5% (v:v) NP-40 with one tablet of protease inhibitor cocktail (Roche) per 10 ml of buffer), and collected in 1.5 ml microcentrifuge tube and rocked at 4°C for 30 min. Cell Lysates were sedimented at 16,000 *g* for 10 min at 4°C. The supernatants were saved as the NP-40-soluble fraction. Pellets were washed once with lysis buffer and solubilized with 2×SDS buffer (125 mM Tris·HCl, 4% SDS, 5% β-mercaptoethanol, 20% glycerol, 0.01% bromophenol blue) and saved as the NP-40-insoluble fraction [17].

### Lectin Binding Assay

Presence and linkage patterns of terminally linked sialic acids on SP-A2 proteins expressed in CHO-K1 cells were detected with digoxigenin-labeled lectins according to manufacturer’s instruction (DIG Glycan Differentiation Kit, Roche Applied Science, Indianapolis, IN). Briefly, the secretory SP-A2 from the medium were immunoprecipitated with V5 antibody, then analyzed by reducing SDS-PAGE and Western blotting. After transfer, the lectins *Maackia amurensis agglutinin* (MAA) and *Sambucus nigra agglutinin* (SNA) were incubated with the NC membrane to detect terminal *α*(2,3)-linked and *α*(2,6)-linked sialic acids, respectively. Both positive and negative control glycoproteins were included to assess lectin binding efficiency and specificity.

### Immunofluorescence Microscopy

CHO-K1 cells were culture on the coverslip coated with poly-lysine (Sigma) at 150,000 cells per 35 mm dish. Twenty-four hours after transfection, the cells were rinsed twice with cold PBS, fixed by 4% paraformaldehyde (PFA), permeabilized with 0.1% Triton at RT for 5 min and blocked with chicken serum for 30 min at RT. V5-tagged SP-A2 was detected using anti-V5 antibody overnight at 4°C. Calreticulin antibody was used to stain endoplasmic reticulum. Secondary antibody staining was performed using a commercial goat anti-mouse IgG Alexa-488 conjugate or goat anti-rabbit IgG Alexa-594 conjugate (Molecular Probes). Experiments were performed at least in triplicate and representative results are shown. Cell images were obtained by Olympus FV1000 confocal microscope.

### Chemical Chaperone Treatment

Transfected CHO-K1 cells were treated with 4-phenlybutrate acid (Sigma) in DMEM culture media at 37°C for 36–48 h. The conditional medium and NP-40-soluble and -insoluble fractions were analyzed by western blotting.

### Statistical Analysis

Statistical analyses were performed using Prism (GraphPad Software, Inc., La Jolla, CA), data are expressed as mean ± S.D. The statistical significance of the differences between the means of groups was determined by unpaired two-tailed t-tests; a value of P<0.05 was considered significant.

## Results

### SP-A2 Mutations in CRD Domain Impair Protein Secretion in CHO-K1 Cells

**Figure 1:**
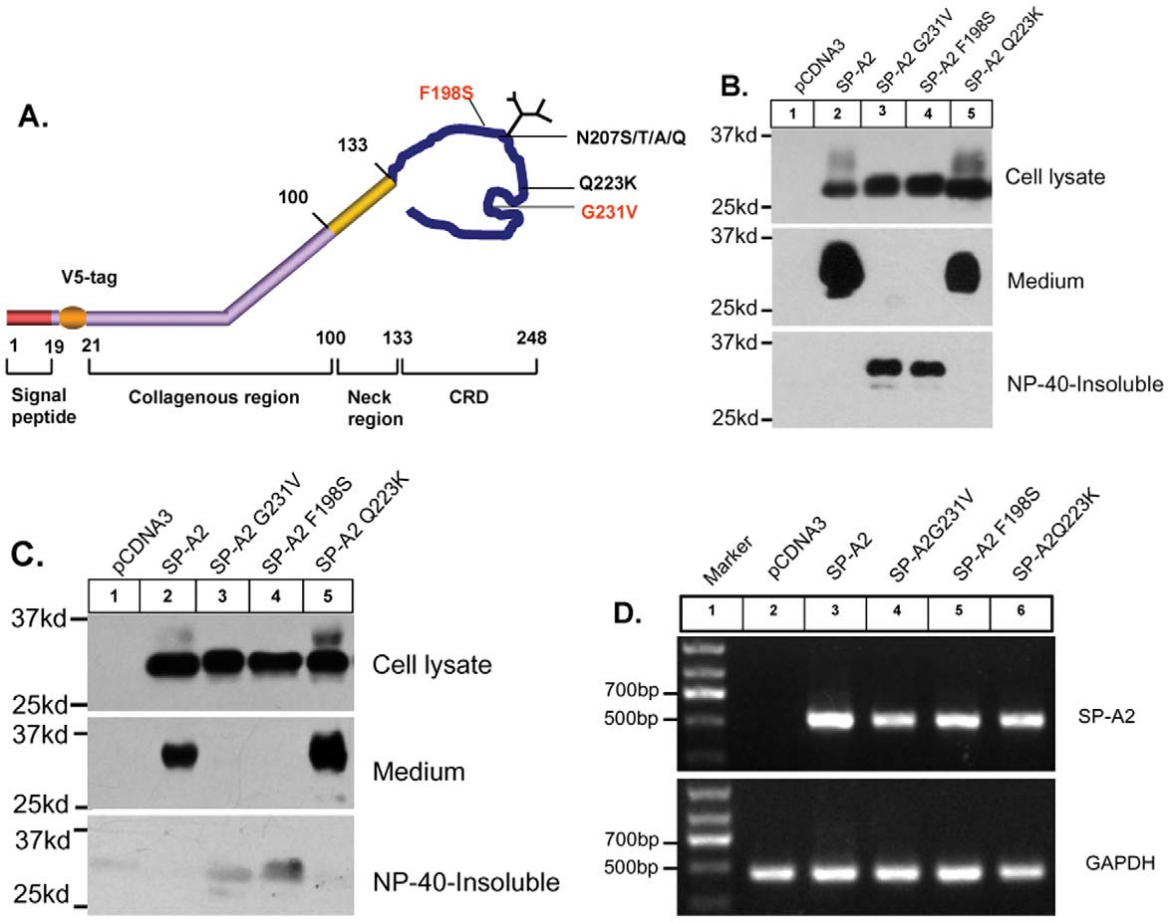
Expression of SP-A2 wild-type and mutant protein and mRNA in CHO-K1 cells. (**A**) SP-A2 schematic. SP-A2 is tagged with V5 epitope at amino acid position 21 follow the signal sequence, four functional domains are indicated, and various mutations are also listed. **CRD**, carbohydrate recognition domain. (**B**) CHO-K1 cells were transiently transfected with vector (pcDNA3.0), V5-tagged SP-A2 wild-type, G231V, F198S and Q223K variants full-length cDNA plasmids. Forty-eight hours after transfection, equal amounts of total protein from cell lysate or media were subjected to SDS-PAGE and followed by immunoblot analysis using a monoclonal antibody that recognizes the V5 epitope. (**C**) Stably expressing vector, V5-tagged SP-A2 wild-type, G231V, F198S and Q223K variants in CHO-K1 cells. 5×10^5^ cells/well were seeded and cultured in 6-well plate for 48 h, cell lysate and medium were collected and analyzed by SDS-PAGE and western blotting. (**D**) CHO-K1 cells were transiently transfected with plasmids pcDNA3.0, V5-tagged SP-A2 wild-type, G231V, F198S and Q223K variants. Forty-eight hours later, total RNA was extracted and RT-PCR was performed using primers specific to SP-A2 and GAPDH gene.

It had been reported that the pulmonary fibrosis associated SP-A2 mutations (G231V and F198S) can result in a deficiency in protein secretion in both A549 cells and type II alveolar epithelial cells [16]. To further explore the potential mechanism, we transiently transfected constructs expressing V5-tagged wildtype, G231V, F198S, and Q223K SP-A2 in CHO-K1 cells (Fig. 1A). The protein expression and secretion was confirmed in these cells and is shown in Figure 1B. The wild-type SP-A2 protein from cell lysate showed two major bands. The lower band at 28 kDa has a higher density compared to the upper band at 32–36 kDa. This upper band is not observed in A549 cell lysates [16], and has the same molecular weight as the secreted form of the protein (Fig. 1B, top panel, lane 2). Expression of SP-A2 Q223K, which is a common variant, was very similar to wild-type SP-A2 (Fig. 1B, top panel, lane 5). Interestingly, only the lower molecular weight SP-A2 protein was observed in the CHO-K1 cell lysate of the G231V and F198S mutants (Fig. 1B, top panel, lane 3, 4). Consistent with that, the secreted form of SP-A2 was not detected in the G231V and F198S mutants but was observed for both the wild-type and Q223K expressing cells (Fig. 1B, middle panel).

We established CHO-K1 cell lines which stably express wild-type, G231V, F198S, and Q223K SP-A2 protein (Fig. S1). The protein expression of wild-type and the mutatns of SP-A2 in the cell lysate and medium of these cells show very similar patterns to those observed for the transiently transfected cells (Fig. lC). This result suggests that the SP-A2 G231V, F198S mutations might cause the observed posttranslational modification and secretion deficiencies.

To examine the effect of the mutation of SP-A2 on the mRNA transcription, total RNA was extracted from CHO-K1 cells 48 h after transient transfection of wild-type and mutant SP-A2 constructs. SP-A2 and GAPDH primers were used to amplify their corresponding mRNA by RT-PCR. No endogenous SP-A2 mRNA was detected in CHO-K1 cells transfected with vector (pcDNA 3.0), while similar levels of SP-A2 mRNA expression were shown in cells transfected with either wild-type or different mutant constructs (Fig. 1D).

### SP-A2 Mutations Impair Dimmer/trimer Formation Contributing to Protein Secretion Deficiency

**Figure 2:**
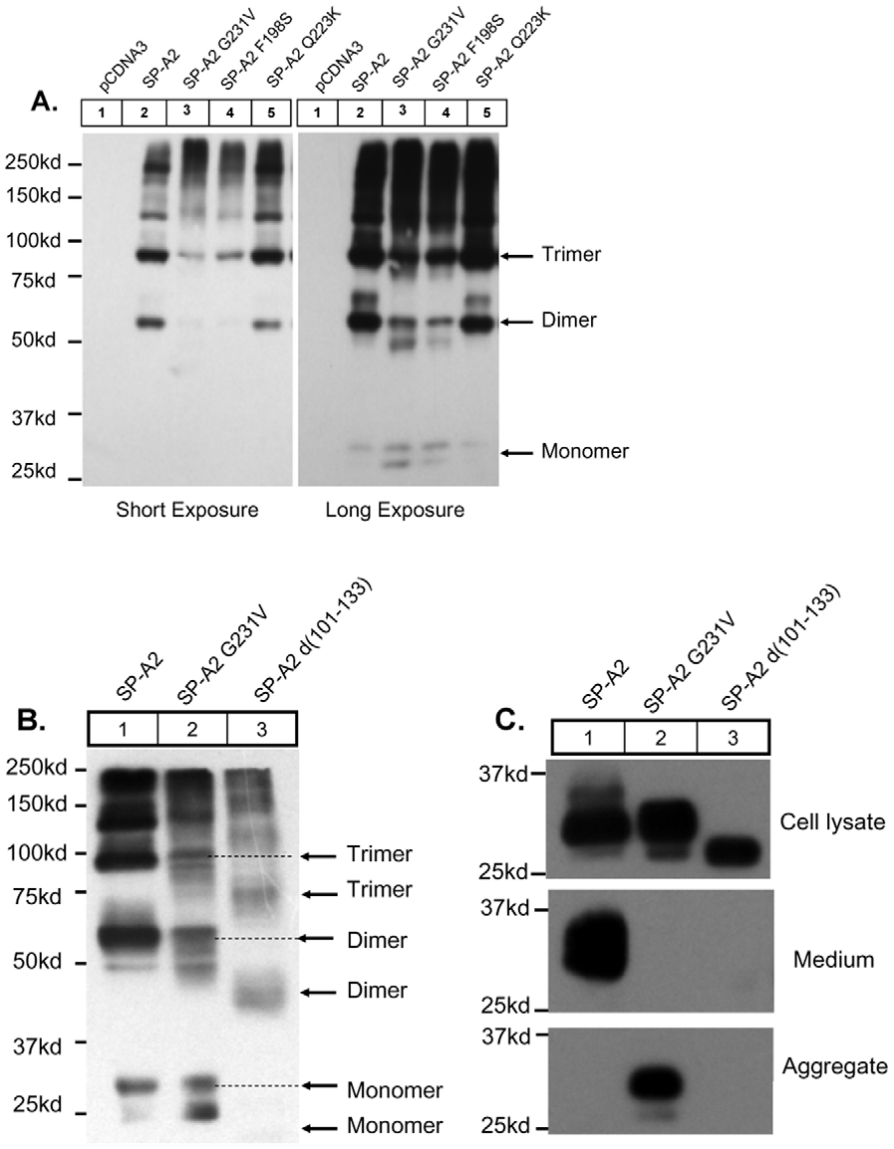
SP-A2 mutations impair dimmer/timer which contributes to protein secretion deficiency. (**A**) CHO-K1 Cells were transiently expressed vector (pcDNA3.0), SP-A2 wild-type, G231V, F198S, Q223K plasmids, 48 h after transfection the cells were harvested and the protein was analysed by SDS-PAGE and western blotting under nonreduced condition without β-mercaptoethanol. (**B,C**) Transiently expression of SP-A2 d(100–133), a neck region deletion mutation, in CHO-K1 for 48 h, the cells were lysated and separated by SDS-PAGE and western blotting under nonreduced condition (**B**) or reduced condition (**C**, upper panel). SP-A2 d (100–133) mutation can block dimmer/trimer formation (**B**) and protein secretion (**C,** middle panel). Monomer, dimmer, and trimer complexes are indicated.

It has been suggested that the trimerization of SP-A is very important for protein secretion [18], [19], [20]. We next examined the protein oligomerization by SDS-PAGE under non-reducing condition in CHO-K1 cells. Compared to wild-type, the SP-A2 G231V and F198S mutants have much lower density at the expected size of the dimeric SP-A2, ∼60 kDa, and trimeric SP-A2, ∼90 kDa (Fig. 2A). This suggests that the impairment in the formation of the dimmer/trimer in the mutant SP-A2 might contribute to the deficiency in protein secretion. To test this hypothesis, we generated a construct of SP-A2 with deletion of the neck region (SP-A2 d (100–133)). This deletion results in the loss of dimmer/trimer formation [18]. When this construct is expressed in CHO-K1 cells, we confirmed the impairment of dimmer/trimer formation under non-reducing conditions. In addition, we also observed that the protein secretion in these cell lines was completely blocked (Fig. 2B and C).

### Impairment of Dimmer/trimer Leads to SP-A2 Mutant Protein Sialylation Deficiency, but not N-linked Glycosylation Deficiency

**Figure 3:**
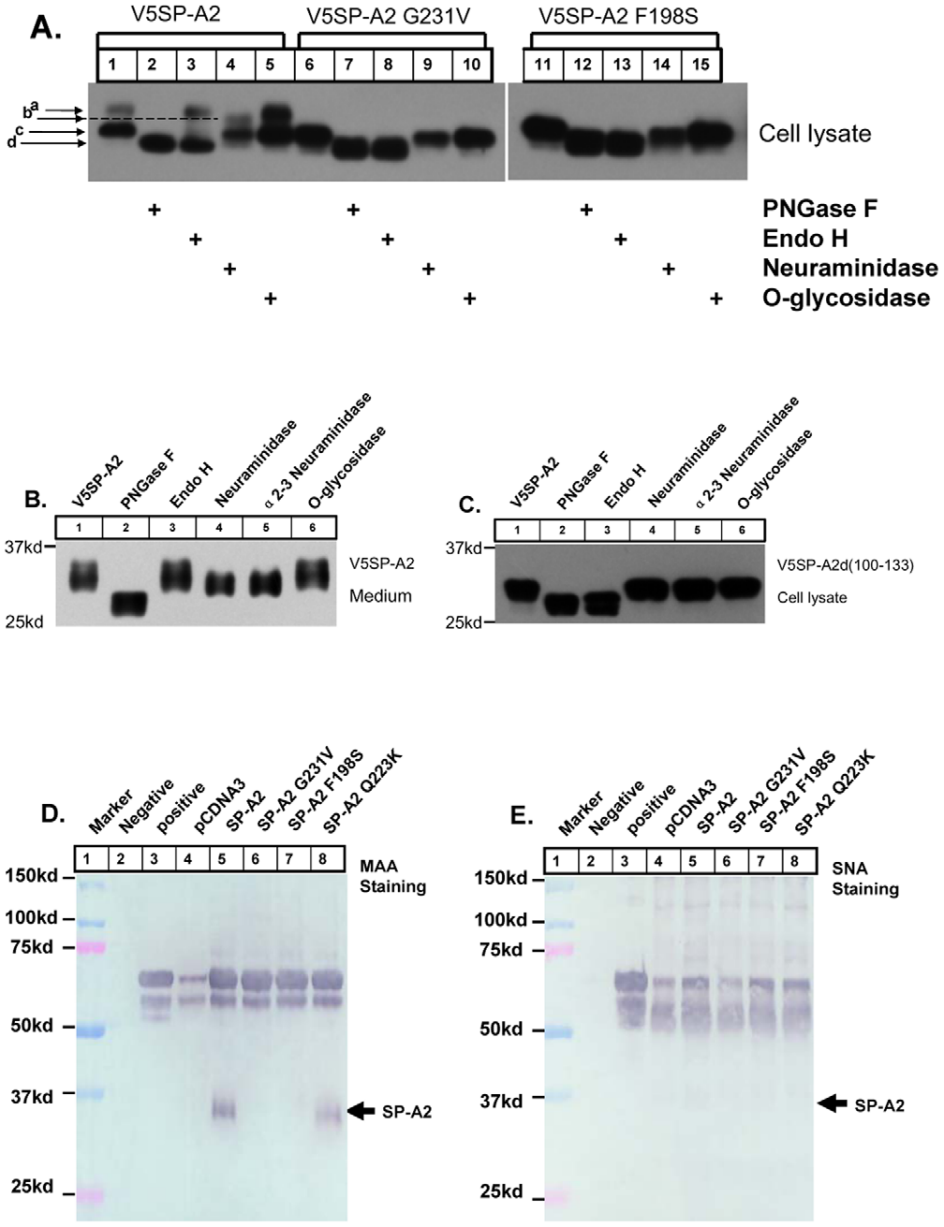
Analysis of glycosylation modifications of human SP-A2 in cell lysate and medium. CHO-K1 Cells were transiently expressed SP-A2 wild-type, G231V, F198S or d (100–133) plasmids for 48 h and the cell lysate (**A and C**) or medium (**B**) were treated without or with (+) PNGase F, Endo H, Neuraminidase, O-glycosidase as described in Materials and methods. Digestion products were subjected to SDS-PAGE, transferred to NC membranes, immunoblotted with anti-V5 antibody. **a**.Mature and sialylated SP-A2 protein. **b.** Desialylated SP-A2. **c.** Immature N-glycosylated SP-A2, but not sialylated. **d.** Deglycosylated SP-A2. Presence and linkage patterns of terminally linked sialic acids on mature secreted SP-A2 from medium were further analyzed by immuoprecipitation and Western blotting, followed by detection with digoxigenin-labeled lectins and anti-digoxigenin-alkaline phosphatase system (**D and E**). To assess lectin binding efficiency and specificity both positive control glycoproteins (fetuin for *α*(2,3)-linkage and transferrin for *α*(2,6)-linkage) and negative control (N-Glycosidase F treated transferrin) were included.

It has been suggested that glycosylation of SP-A is important for secretion of the protein [3]. To explore whether glycosylation contributes to impairment of dimmer/trimer formation and SP-A2 secretion, we used various glycosidase including PNGase F, endoglycosidase H (Endo H), neuraminidase (sialidase) and O-glycosidase, to identify the modifications present in wild-type SP-A2. PNGase F treatment results in the remove of all N-linked sugars while Endo H removes high-mannose sugars from protein that has not yet undergone full maturation. Neuraminidase and O-glycosidase will remove sialic acid and O-linked sugars, respectively. Fig. 3A shows that the major lower protein band from the cell lysate was sensitive to PNGase F and EndoH (lane 2, 3) but not to neuraminidase and O-glycosidase (lane 4, 5). The top protein band was sensitive to PNGase F and neuraminidase (lane 2, 4) but not to Endo H and O-glycosidase (lane 3, 5). These results indicated that the low band was N-linked glycosylated but not O-linked glycosylated, while the higher molecular weight band is sialylated and this band represents the fully glycosylated form of the protein. Our results are consistent to Whitsett’s work that showed wild-type SP-A was quickly processed to an Endo H-sensitive form and then slowly sialylated prior to secretion from type II alveolar epithelial cells [3]. The mutant SP-A2 G231V and F198S proteins expressing cells show a single SP-A2 band and the glycosidase digestion pattern is identical to that of lower protein band of wild-type SP-A2 (lane 6–15). This result demonstrates that SP-A2 G231V and F198S mutants can be N-linked glycosylated, but are not further sialylated. In addition, the digestion pattern of mature secreted wild-type SP-A2 from the medium was determined to be the same as the upper protein band from the cell lysate (Fig. 3B). Meanwhile, the sensitivity of mature SP-A2 to α2–3 neuraminidase indicates that this form of the protein contains α2–3 sialylation (Fig. 3B, lane 5). Indeed, using lectin binding assay, we found that only mature secreted wildtype and Q223K SP-A2 could bind to MAA (Fig. 3D, lane 5 and 8), but could not bind to SNA (Fig. 3E). The result further confirmed the mature SP-A2 is α2–3 sialylated but not α2–6 sialylated. Importantly, when expressed in CHO-K1 cells, the SP-A2 d(100–133) mutant protein which lacks the dimmer/trimer assembly, displays similar glycosidase digestion patterns as the G231V and F198S mutant SP-A2 protein (Fig. 3C). All these results taken together suggest that the impairment of the dimmer/trimer formation in the SP-A2 mutant proteins does not affect the N-linked glycosylation but does cause sialylation deficiency.

### Inhibition of Glycosylation did not Completely Block SP-A2 Protein Secretion

**Figure 4:**
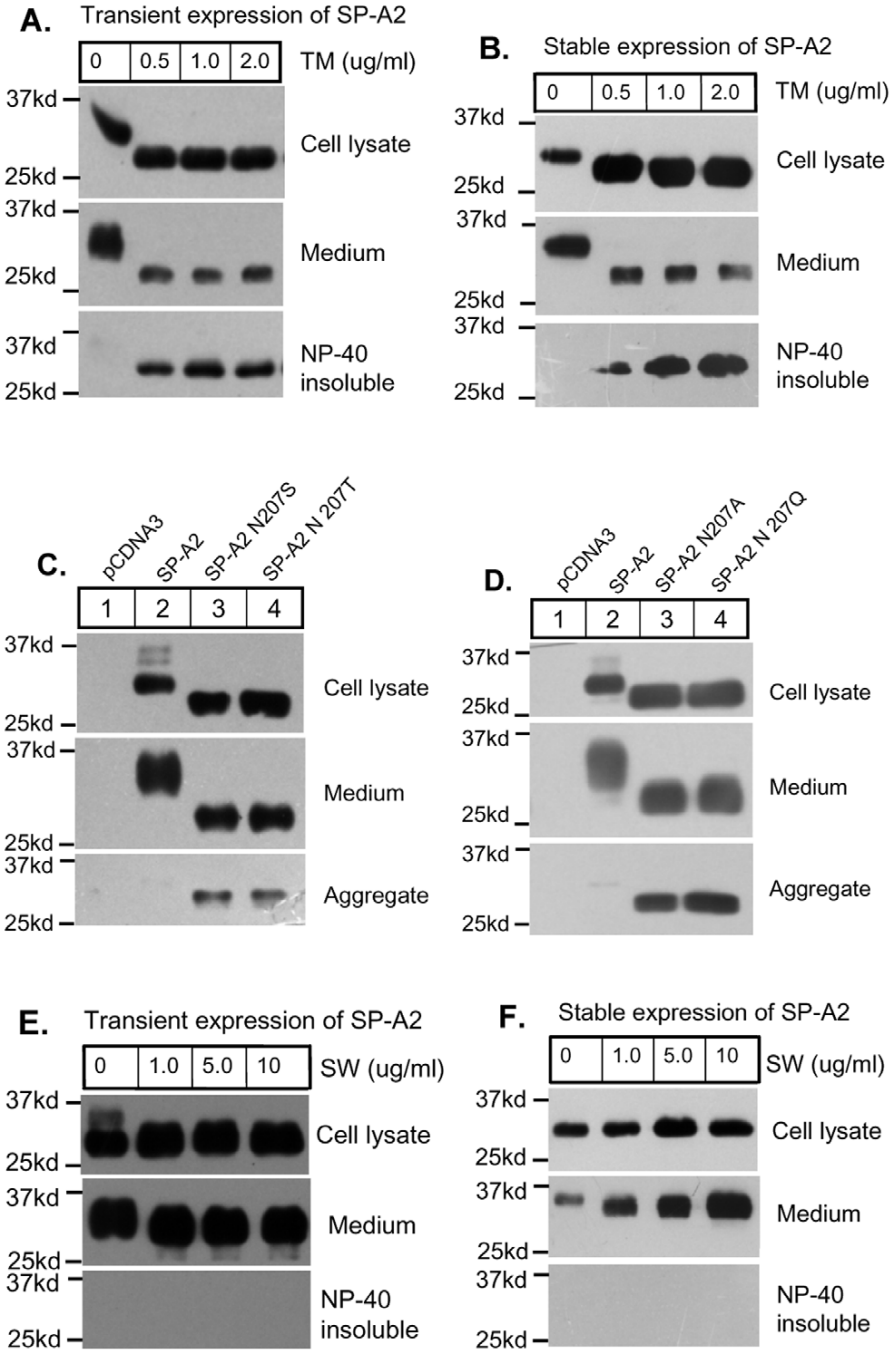
Blocking of glycosylation or sialylation did not completely impair SP-A2 protein secretion. (**A–B**) CHO-K1 cells which were transient (**A**) or stable (**B**) expression of wild-type SP-A2 were treated with tunicamycin (TM) for indicated concentration, medium and cell lysate were harvested and separated by SDS-PAGE and immunoblotted with anti-V5 antibody. Tunicamycin could not completely block SP-A2 secretion, but causes NP-40 insoluble aggregates. (**C–D**) SP-A2 N207S/T/A/Q, the glycosylated site mutated SP-A2 plasmids, were transfected into CHO-K1 cells for 48 h, cell lysate, medium and aggregates were harvested and separated by SDS-PAGE and immunoblotted with anti-V5 antibody to check protein expression, secretion and aggregation. (**E–F**) CHO-K1 cells which were transient (**E**) or stable (**F**) expression of wild-type SP-A2 were treated with swainsonine (SW) for indicated concentration, lysate, medium and aggregates were detected by immunoblotting.

To further explore the relationship between SP-A2 glycosylation and protein secretion, we treated the transiently wild-type SP-A2 transfected CHO-K1 cells with tunicamycin, an inhibitor of protein N-linked glycosylation. We found that even low concentrations (0.5 µg/ml) of tunicamycin can completely block wild-type SP-A2 N-linked glycosylation. Surprisingly, unglycosylated SP-A2 can be detected in the medium, although at a lower abundance compared to cell not treated with tunicamycin (Fig. 4A). Similar results were achieved when we treated the stably expressing SP-A2 cell lines with tunicamycin (Fig. 4B). These results indicate that N-linked glycosylation was not necessary for SP-A2 secretion. To further confirm this result, we generated two glycosylation-defective mutants, SP-A2 N207S and N207T (Fig. 1A), from which expressed protein could not be N-linked glycosylated (Fig. S2). When we transiently expressed these mutants in CHO-K1 cells, the unglycosylated SP-A2 is also detected in the cell lysates as well as in the medium, although the amount of secreted protein was reduced compared to that of wild-type (Fig. 4C). Two more glycosylation-defective mutants, SP-A2 N207A and N207Q, also show similar results (Fig. 4D).

We also treated the CHO-K1 cells either transiently or stably expressing wild-type SP-A2 with swaisonine, an inhibitor of α-mannosidase II, and found that swaisonine could block synthesis of fully sialylated SP-A2 and resulted in secretion of partially glycosylated SP-A2 (Fig. 4E and F, middle panel). All the above results indicate that the glycosylation of SP-A2 are not necessary for protein secretion.

### Mutant SP-A2 with Secretion Deficiency is Retained in the ER

**Figure 5:**
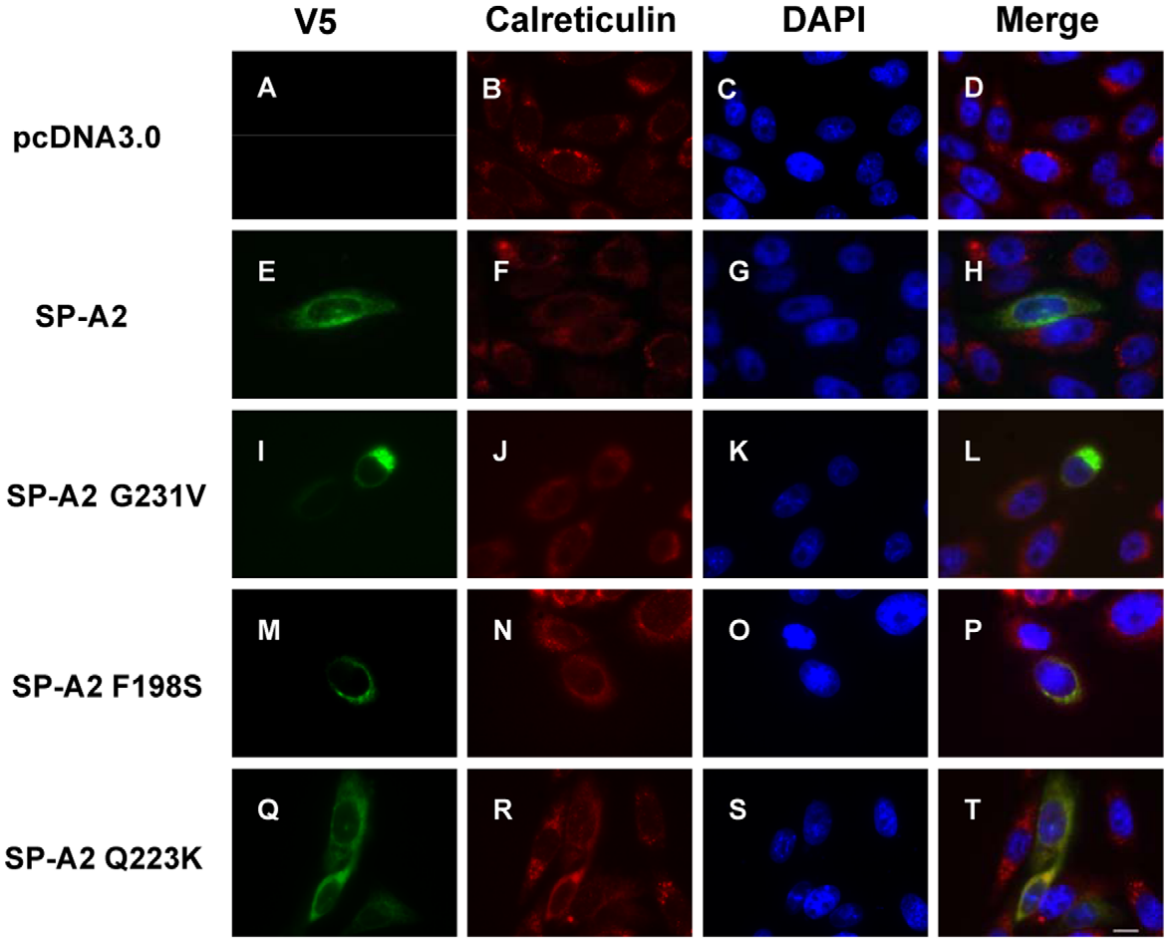
Intracellular localization of SP-A2 wild-type and variants. CHO-K1 Cells were transiently transfected with SP-A2 wild-type, G231V, F198S, Q223K plasmids, 48 h after transfection, the cells were fixed by 4% PFA, the localization of SP-A2 was examined by indirect immunofluorescence assay, and images were collected by confocal microscopy as described in Materials and Methods. Wild-type and Q223K distributed evenly in cellular which colocalized in part with calreticulin (ER marker) (**H,T**). Most of G231V, F198S mutants colocalized with calreticulin (**L,P**). Scale bar 10 µm.

Since the mutant SP-A2 does not secret and accumulates in the cell, we wanted to determine the subcellular distribution of wild-type and mutant SP-A2. Transiently transfected CHO-K1 cells were examined by confocal laser scanning microscope. We used anti-V5 monoclonal antibody and anti-calreticulin polyclonal antibody to locate V5 tagged SP-A2 and the ER, respectively. The control cells transfected with pcDNA3.0 did not show any V5 staining (Fig. 5A–D). Wild-type and Q223K SP-A2 distributes in the cytoplasm evenly and partially co-localizes with calreticulin, a marker of the ER (Fig. 5 E–H, Q–T). However, G231V and F198S SP-A2 mutants distribute in the pre-nuclear region and almost completely co-localizes with calreticulin (Fig. 5I-L, M-P). This indicates both mutant proteins are retained in the ER and this result is consistent with a previous report [16].

### SP-A2 Mutant Proteins are Degraded by Proteosome Pathway but not Lysosome or Autophagy Pathway

**Figure 6:**
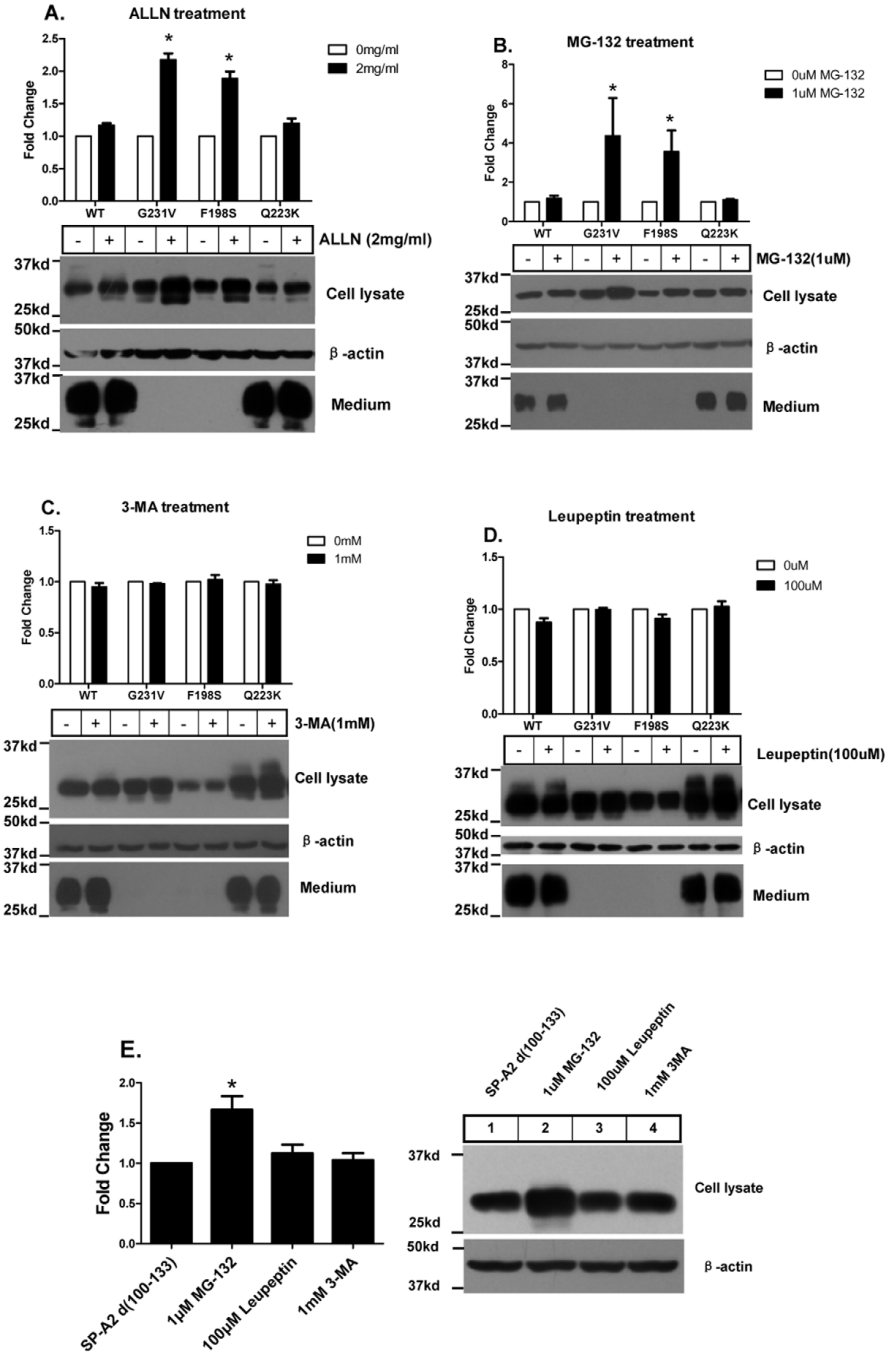
SP-A2 G231V, F198S proteins partially degraded through proteasome pathway but not lysosome or autophagy pathway. (**A–D**) CHO-K1 Cells were transiently expressed SP-A2 wild-type, G231V, F198S, Q223K plasmids, 24 h later change medium and treated the cell with ALLN (**A**), MG-132 (**B**), 3-MA (**C**), leupeptin (**D**) for 24 h, then cell lysate and medium were collected, analyzed by SDS-PAGE and western blotting. (**E**) SP-A2 Neck region (100–133) mutant expression is increased by inhibition of proteasome pathway but not lysosome or autophagy pathway. The amount of each SP-A variant was normalized to its expression in the absence of inhibitors. Data are shown as the mean±S.D. for at least three independent transfection experiments.

The accumulated mutant proteins in the cells may be either subject to degradation or may form aggregates. To investigate whether mutant SP-A2 is degraded, we inhibited three major degradation pathways including the proteasome, the lysosome, and the autophagy pathways. After CHO-K1 cells were transiently transfected with wild-type or mutant SP-A2 for 6 h, N-Acetyl-Leu-Leu-Norleual (ALLN), an inhibitor of the proteasome, was added to the culture for 48 h and the SP-A2 proteins from the cell lysate and the medium were detected by immunoblotting. Wild-type and Q223K SP-A2 proteins in the cell lysate and medium did not show any changes after ALLN treatment. The G231V and F198S mutant SP-A2 protein expression level in the cell lysate was significantly increased by 2.2 and 1.9 fold respectively while the protein from the medium was still undetectable after ALLN treatment (Fig. 6A). We used another proteasome inhibitor MG-132 to treat the cells and these experiments gave similar results (Fig. 6B). This data indicates that these two mutants G231V and F198S can be degraded through the proteasome pathway. The lysosome and autophagy pathways are not involved in G231V and F198S SP-A2 degradation because the treatments with the lysosome inhibitor Leupeptin or the autophagy inhibitor 3-Methylamphetamine (3-MA) does not alter protein expression (Fig. 6C and D). Moreover, we also found that intracellular accumulated SP-A2 d (100–133) can also be degraded through the proteasome pathway (Fig. 6E). It should also be noted that the inhibitors of these degradation pathways do not affect wild-type or mutant SP-A2 protein secretion. Thus, these data suggest that mutant SP-A2 is degraded via proteasome pathway in CHO-K1 cells.

### Proteasome Inhibitor Increases the Aggregates of Mutant SP-A2 Proteins

**Figure 7:**
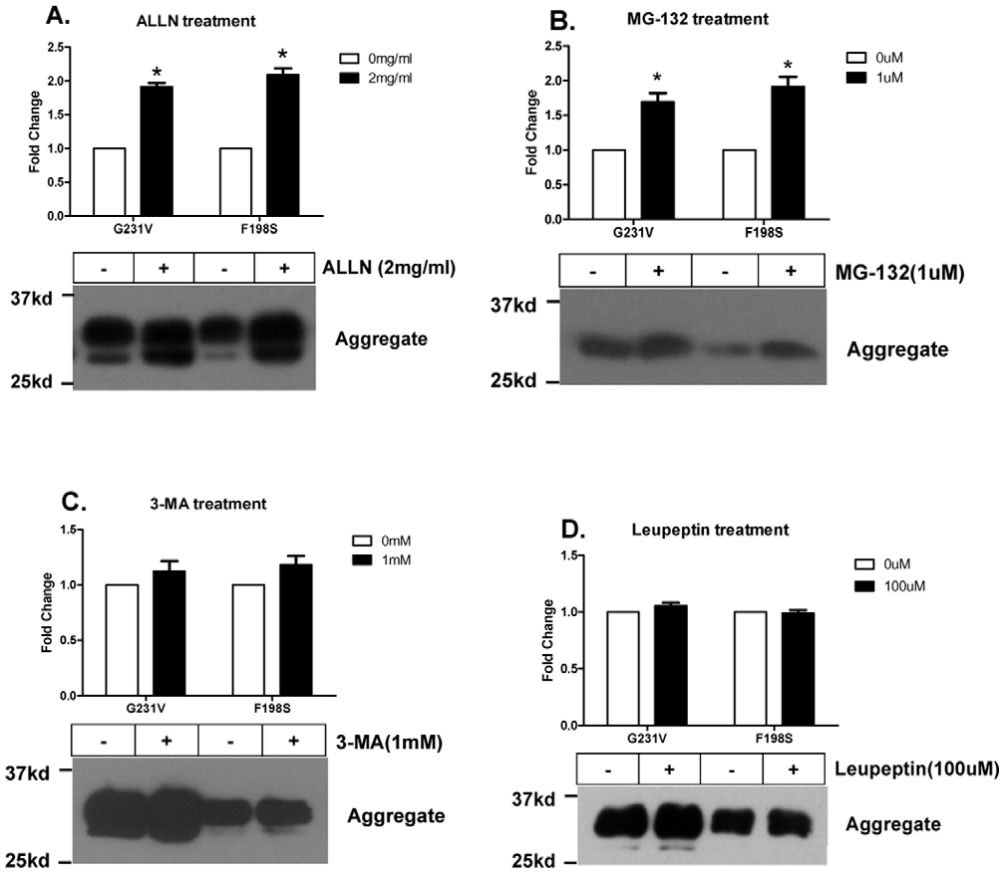
Proteasome inhibitor increases the aggregates of mutant SP-A2 proteins. CHO-K1 Cells were transiently expressed SP-A2 wild-type, G231V, F198S,Q223K plasmids, 24 h later change medium and treated the cell with ALLN (**A**), MG-132 (**B**), 3-MA (**C**), leupeptin (**D**) for 24 h, then the NP-40-insoluble aggregates were analyzed by SDS-PAGE and western blotting. The amount of each SP-A variant was normalized to its expression in the absence of inhibitors. Data are shown as the mean±S.D. for at least three independent transfection experiments.

Next we examined the aggregate formation in the G231V and F198S SP-A2 expressing CHO-K1 cells. We segregated the NP-40-soluble and -insoluble fractions from cell lysate by centrifugation. The G231V and F198S SP-A2 expressing cells do contain NP-40-insoluble aggregates in both transiently and stably expressing CHO-K1 cells while wild-type and Q223K SP-A2 expressing cells do not (Fig. 1B and C, bottom panel). Intriguingly, ALLN or MG-132 treatment of the cells increases the aggregate accumulation in the G231V or F198S SP-A2 transfected cells, while 3-MA or leupeptin treatment does not, confirming that the mutants SP-A2 can be degraded via the proteasomal pathway in CHO-K1 cells (Fig. 7A–D). We also found that tunicamycin treatment causes wild-type SP-A2 aggregation in CHO-K1 cells with stably or transiently expressed SP-A2 (Fig. 4A and B, bottom panel). Meanwhile, transfection with the glycosylation-defective mutant SP-A2 (N207S, N207T, N207A, N207Q) constructs also caused aggregate formation (Fig. 4C and D, bottom panel), confirming the inhibition of N-linked glycosylation of SP-A2 also induces protein aggregation. However, blocking sialylation of SP-A2 with swainsonine does not lead to SP-A2 aggregates (Fig. 4E and F, bottom panel).

### 4-PBA Partially Alleviates Protein Aggregate and Rescues the Secretion of Mutant SP-A2

**Figure 8:**
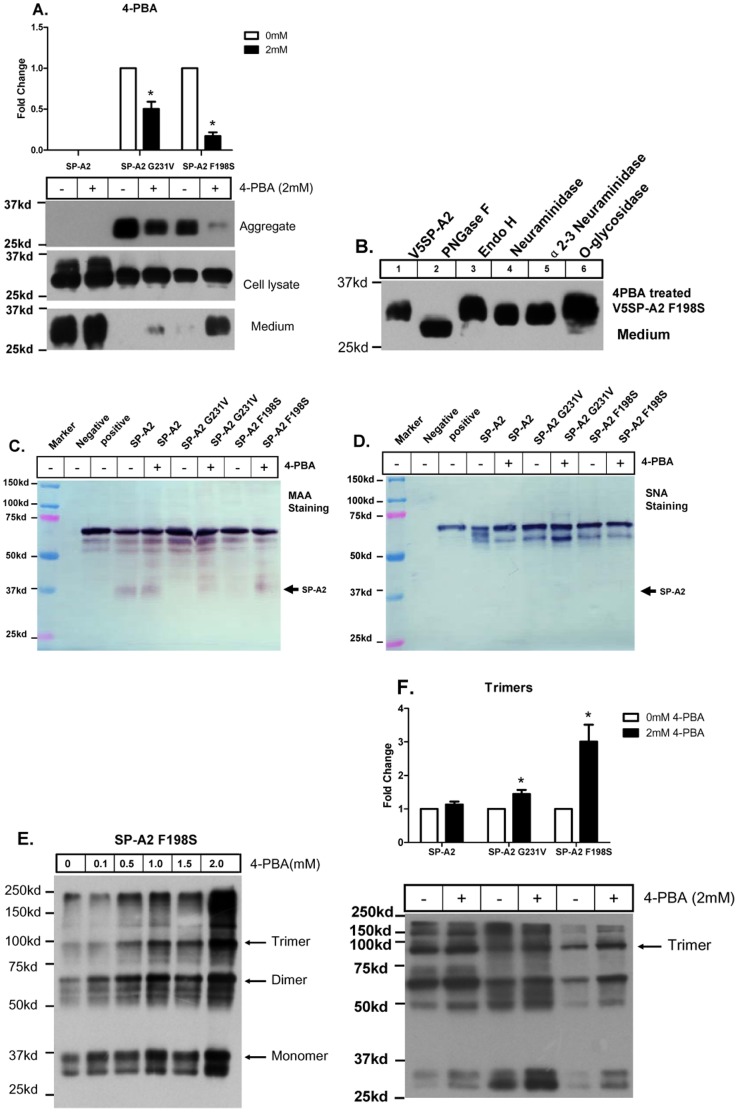
Chemical chaperone partially alleviates the mutant protein aggregates and rescues secretion. CHO-K1 cells were transiently transfected wild-type, G231V and F198S variants, 24 h later changed the medium and incubated 4-PBA (2 mM) for 36–48 h. The NP 40-insoluble and -soluble fractions from cell lysate, and the proteins in the medium were subjected to SDS-PAGE, transferred to NC memberane, and immunoblotted with antibody against the V5 epitope. The NP-40-insoluble aggregates were quantitated by Image J software. The amount of each SP-A variant was normalized to its expression in the absence of 4-PBA. Data are shown as the mean±S.D. for three independent transfection experiments (**A**). Presence and linkage patterns of terminally linked sialic acids on mutant secreted SP-A2 from medium after 4-PBA treatment were analyzed by various glycosylase (**B**) and sialylation modification was further confirmed by specific lectin binding assay, MAA and SNA specificly bind to*α*(2,3)-linkage (**C**) and*α*(2,6)-linkage (**D**) sialic acid respectively. The positive control glycoproteins (fetuin for *α*(2,3)-linkage and transferrin for *α*(2,6)-linkage) and negative control (N-Glycosidase F treated transferrin) were included. Nonreducing gel were used to identify SP-A2 dimmer/trimer after 4-PBA treatment (**E, F**).

The aggregate formation observed in the G231V and F198S SP-A2 expressing cells may be due to protein misfolding, as reported in some other disease-causing mutations [21]. To test whether the G231V and F198S mutations affect SP-A2 structure and function, two prediction software programs Polyphen (http://genetics.bwh.harvard.edu/pph/) and SIFT (http://sift.bii.a-star.edu.sg/) were used and both of these programs predict that the G231V and F198S mutations can cause protein damage. It has been reported that chemical chaperones, a kind of small molecules, can mimic the function of intracellular molecular chaperones which stabilize mutant proteins and facilitate their proper folding. We utilized one such molecular chaperone 4-phenylbutyric acid (4-PBA) to test whether a chaperone can rescue the misfolded protein. After a 48 h 4-PBA treatment of CHO-K1 cells, which were transiently transfected with mutant SP-A2, the aggregates of G231V and F198S SP-A2 were reduced (Fig. 8A, top panel) and this reduction appears to occur in a dose-dependent pattern (Fig. S3A and B). Meanwhile, it appears from the western blot analysis of the medium that 4-PBA treatment partially rescues the secretion of the G231V and F198S mutant SP-A2 protein (Fig.8A, bottom panel). Furthermore, we found that the secreted mutant SP-A2 F198S protein, which dramatically increases with the treatment of the chemical chaperones, were fully sialylated (Fig.8B.). And this modification is α2–3 sialylated but not α2–6 sialylated (Fig. 8C and D), as the same as the mature wildtype SP-A2 is. Interestingly, 4PBA treatment also results in a dose-dependent increase in SP-A2 F198S dimmer/trimer formation, which further confirms that the dimmer/trimer formation might contribute to SP-A2 secretion (Fig.8E). 4-PBA treatment slightly increases SP-A2 G231V secretion, and we also observed that SP-A2 G231V trimers are increased after 4-PBA treatment (Fig. 8F).

## Discussion

Surfactant protein A2 plays an important role on lung surfactant metabolism and immunity [22]. Mutation of SP-A2 on the carbohydrate recognized domain (CRD) at G231V and F198S have been associated with familiar pulmonary fibrosis and lead to recombinant protein secretion deficiency in vitro [15], [16]. This study elucidated the underlying mechanism of SP-A2 secretion deficiency and the subsequently biological effects.

Secretory or membrane proteins are usually translated in the rough ER and then transferred to the Golgi complex for final maturation. Like many secreted proteins, SP-A2 is translated in the lumen of the ER, where the proteins undergo primary N-linked glycosylation. Protein glycosylation is critical to promote proper protein folding, intracellular trafficking, cell surface expression, and secretion [3], [23]. However, our studies reveal that while N-linked glycosylation and sialylation of SP-A2 are not necessary for secretion, the assembly of dimmer/trimer appears to play an important role in SP-A2 secretion. Spissinger T. reported that interfering with the folding and assembly of SP-A by deletion of neck domain resulted in the inhibition of protein secretion [24]. Consistent with our results, the neck domain of human SP-D had been suggested to be necessary for the trimerization of SP-D monomers and then secretion [18]. In addition, the destruction of SP-A trimmers blocks protein secretion [25]. All these evidence supports our hypothesis that the SP-A2 G231V and F198S mutants do not form dimmer/trimer which contributes to the observed secretion and sialylation deficiency. The data from the SP-A2 G231V, F198S and d(100–133) mutants indicate that the monomers of SP-A2 are N-linked glycosylated before they assemble into the dimmer/trimer. We expect that human SP-A2, like rat SP-A in alveolar type II cells [18], can rapidly synthesize 26 kDa nonglycosylated SP-A protein which is then subjected to mannose glycosylation and then can form trimers and higher order oligomers. These proteins are then delivered to the Golgi complex for full sialylation and secretion.

The quality-control system of cells is very important to maintain cell homeostasis. When a misfolded proteins is generated, the cells will either refold or eliminate the protein by degradation through the ubiquitin-proteasome, lysosome or autophagy pathway [25], [26], [27]. We confirmed that a fraction of the mutants SP-A2 is degraded by the proteasome, which plays a strategic role in protecting against the accumulation of abnormal proteins. A defect in this pathway has been related to some forms of interstitial lung disease, such as cystic fibrosis and α1-antitrypsin deficiency [28]. However, when the generation of misfolded proteins exceeds the refolding or degradative capacity of the cell, protein aggregates accumulate [29]. Our results show that proteasome inhibitors increase the amount of cellular mutant proteins which then resulted in more aggregate accumulation (Fig. 7A and B). Studies on mutant Huntington protein also revealed that protein accumulation was significantly increased by the inhibition of the proteasome [30]. All these studies support the mechanism that gene mutation-induced misfolding of a protein can exceed the capacity of the proteasome degradation machinery which results in cytosolic protein aggregation.

Cytosolic aggregation of mutant proteins has been shown to disrupt cellular function. For example, in vitro expression of one of SP-C BRICHOS domain mutation, the exon 4 deletion (hSP-C^Δexon4^), promotes aggregation of protein and causes ER stress, proteasome inhibition, and apotosis in epithelial cells [31]. In addition, previous observations have shown that SP-A2 mutants increased ER stress in A549 cells or primary type II cells [16]. However, more detailed molecular mechanisms are needed to completely understand these mechanisms. The SP-A2 mutations studied here do not inhibit the proteasome, due to the ability of the proteasome to degrade the mutant protein. In addition, it seems that the mechanism of aggregation of mutant SP-A2 is different from that of protein secretion. Because SP-A2 d (100–133) mutation causes a deficiency in secretion but does not induce aggregation (Fig. 2C) by impairing dimmer/trimer assembly. In contrast, when the CHO-K1 cells were treated with tunicamycin, the wild-type SP-A2 formed aggregates but it did not completely block SP-A2 secretion.

Alteration of SP-A secretion has been related to a number of lung diseases [32], including IPF, with which SP-A2 concentration in BAL is relative low [33]. SP-A2 has higher activity to pathogen insult than SP-A1 [13]. Therefore, the SP-A2 G231V or F198S mutation induced protein secretion deficiency would confer lung susceptibility and injury to pathogenic insults. Although SP-A2 mutations seem not to induce apoptosis, they still caused mild ER stress in epithelial cells. So both secretion deficiency and aggregation caused by SP-A2 mutations might contribute to cell injury. At the same time, we speculate that the environmental insults might be needed in collaboration with the cell effects of these mutations to increase lung cell damage and the development to pulmonary fibrosis.

The misfolding and abnormal processing of proteins has been observed in some forms of interstitial lung disease [34]. The SP-A2 mutations resulting in defects in intracellular transport may contribute to the pathogenesis of pulmonary fibrosis. From this view, the inherited pulmonary fibrosis caused by G231V or F198S mutations might be considered as a conformational lung disease. Recently, small chemical molecules named chemical chaperones, such as 4-PBA and trimethylamine oxide (TMAO), have being used to promote protein folding or trafficking. These molecules are being used to develop new therapeutic strategy for conformational diseases [35]. Chemical chaperones are small molecules with chaperone-like activity that can stabilize mutant proteins and reduce their misfolding. 4-PBA is one of chemical chaperones had been approved by US Food and Drug Administration (FDA) for urea-cycle disorders treatment [34]. Intriguingly, in this report, we showed that 4-PBA could partially decrease aggregate formation of the G231V and F198S mutant SP-A2 protein, as well as rescue protein secretion in CHO-K1 cells. It is possible that 4-PBA may increase dimmer/trimer formation of mutant SP-A2 in order to promote protein secretion. Our findings provide possible strategies for prevention of lung malfunction in familial pulmonary fibrosis in the future.

In conclusion, our studies reveal that familial pulmonary fibrosis associated with surfactant protein A2 mutations (G231V and F198S) cause the dimmer/trimer assembly defect and these proteins are retained in the ER. This results in a blocking of protein sailylation and leads to a deficiency in secretion. At the same time, the accumulated mutant proteins in the cells form both soluble and insoluble aggregates that can be degraded through the proteasome pathway. 4-PBA, a chemical chaperone, can partially rescue mutant protein secretion and decrease mutant protein aggregates.

## Supporting Information

Figure S1 Time course of stably expressing vector, V5-tagged SP-A2 wild-type, G231V, F198S and Q223K variants in CHO-K1 cells. Establishment of stably expression SP-A2 wild-type and variants protein in CHO-K1 cells was performed as described in Materials and Methods. After 48 h culture, cell lysate and medium were collected at different time points as shown and analyzed by SDS-PAGE and western blotting.
(TIF)

Figure S2 SP-A2 glycosylated-defective mutants can not be N-linked glycosylated. Cell lysates from CHO-K1 cells transfected with SP-A2 N207S and N207T were digested with PNGase F as described in Materials and Methods.
(TIF)

Figure S3 4-PBA does-dependent decreases NP-40-insoluble aggregates of G231V and F198S variants in CHO-K1 cells. Transient expressing SP-A2 wild-type and variants CHO-K1 cells were incubated with different concentrations of 4-PBA for 48 h and the NP-40-insoluble and -soluble from cell lysate and medium were collected and analyzed by SDS-PAGE and immunoblotting.
(TIF)

Table S1 Primer sequences used in plasmid construction, mutagenesis and RT-PCR.
(DOC)
