# Supplementary figures and images for: Human Surfactant Protein A2 Gene Mutations Impair Dimmer/Trimer Assembly Leading to Deficiency in Protein Sialylation and Secretion
Source: PLoS One. 2012 Oct 3;7(10):e46559. doi: 10.1371/journal.pone.0046559 (PMC3463533; doi:10.1371/journal.pone.0046559)

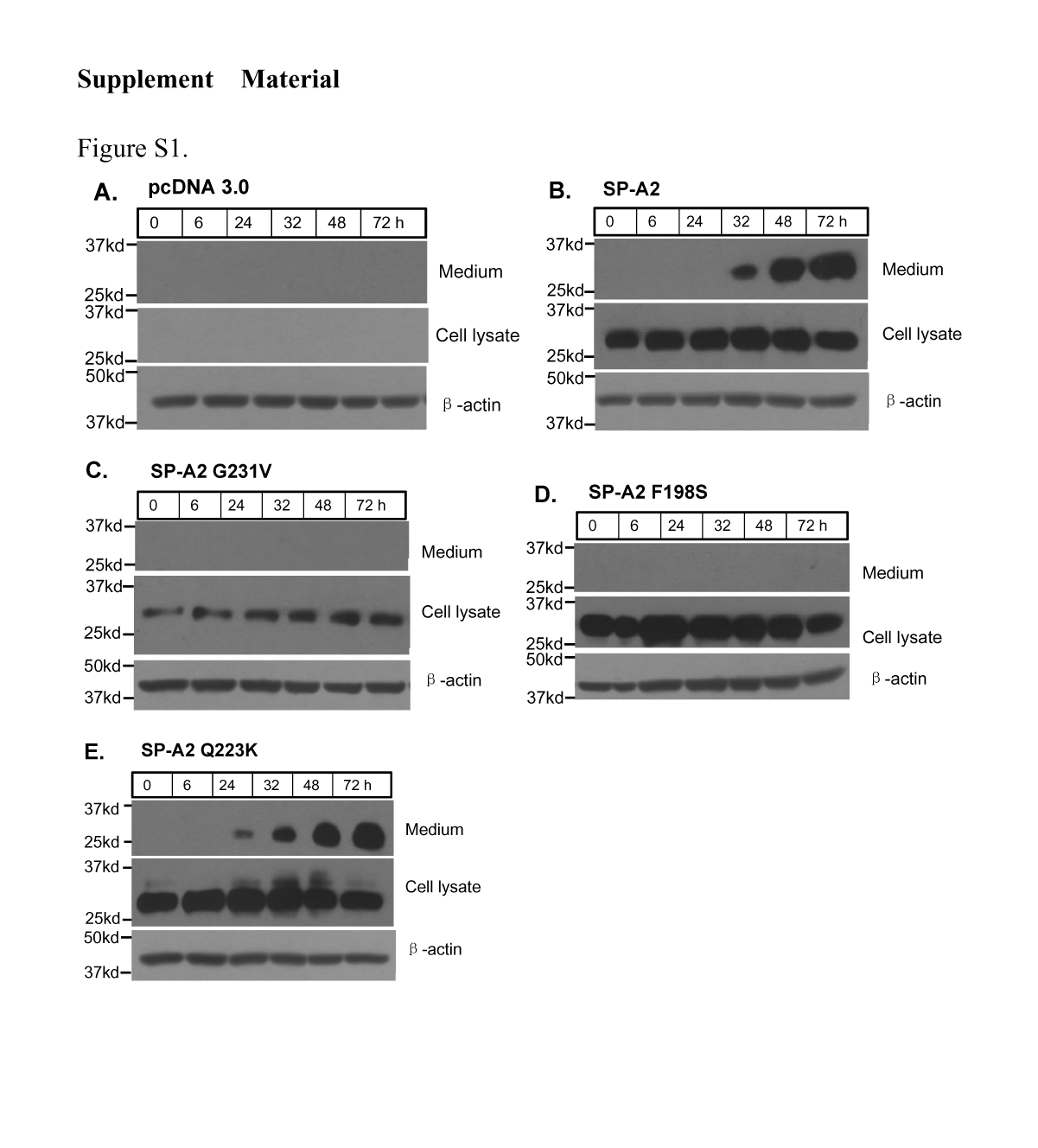

Supplement: Figure S1 — Time course of stably expressing vector, V5-tagged SP-A2 wild-type, G231V, F198S and Q223K variants in CHO-K1 cells. Establishment of stably expression SP-A2 wild-type and variants protein in CHO-K1 cells was performed as described in Materials and Methods. After 48 h culture, cell lysate and medium were collected at different time points as shown and analyzed by SDS-PAGE and western blotting. (TIF) [file pone.0046559.s001.tif]

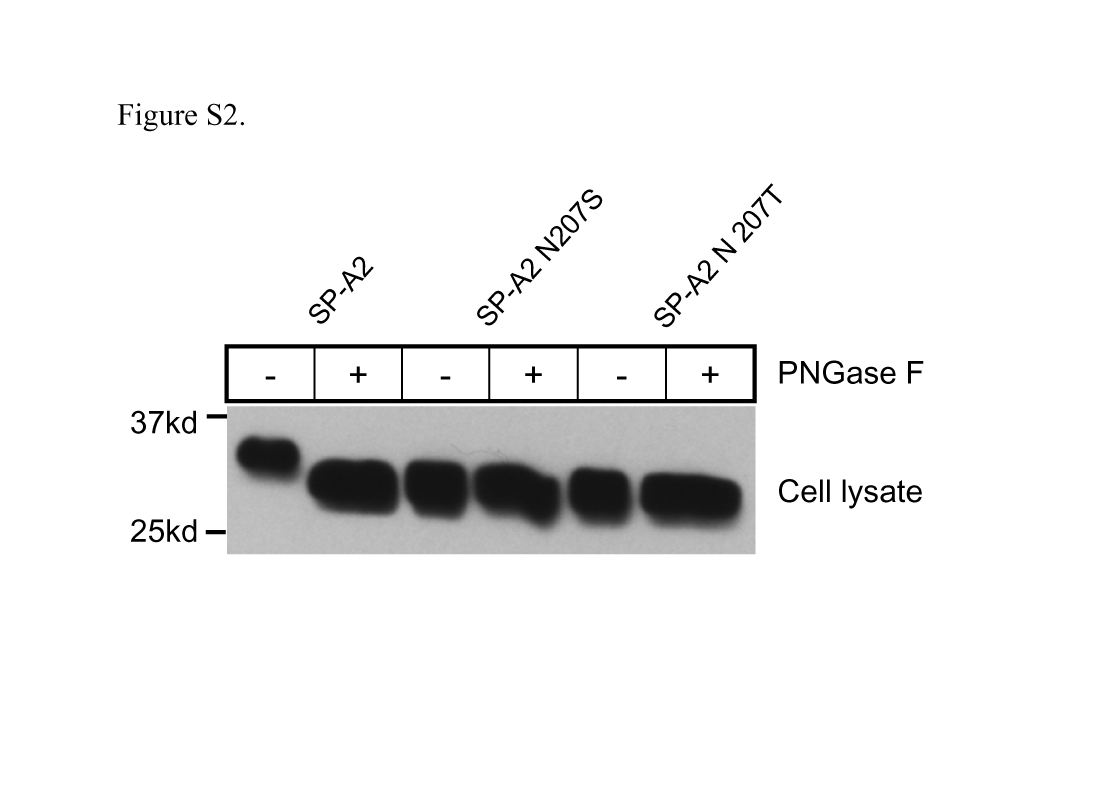

Supplement: Figure S2 — SP-A2 glycosylated-defective mutants can not be N-linked glycosylated. Cell lysates from CHO-K1 cells transfected with SP-A2 N207S and N207T were digested with PNGase F as described in Materials and Methods. (TIF) [file pone.0046559.s002.tif]

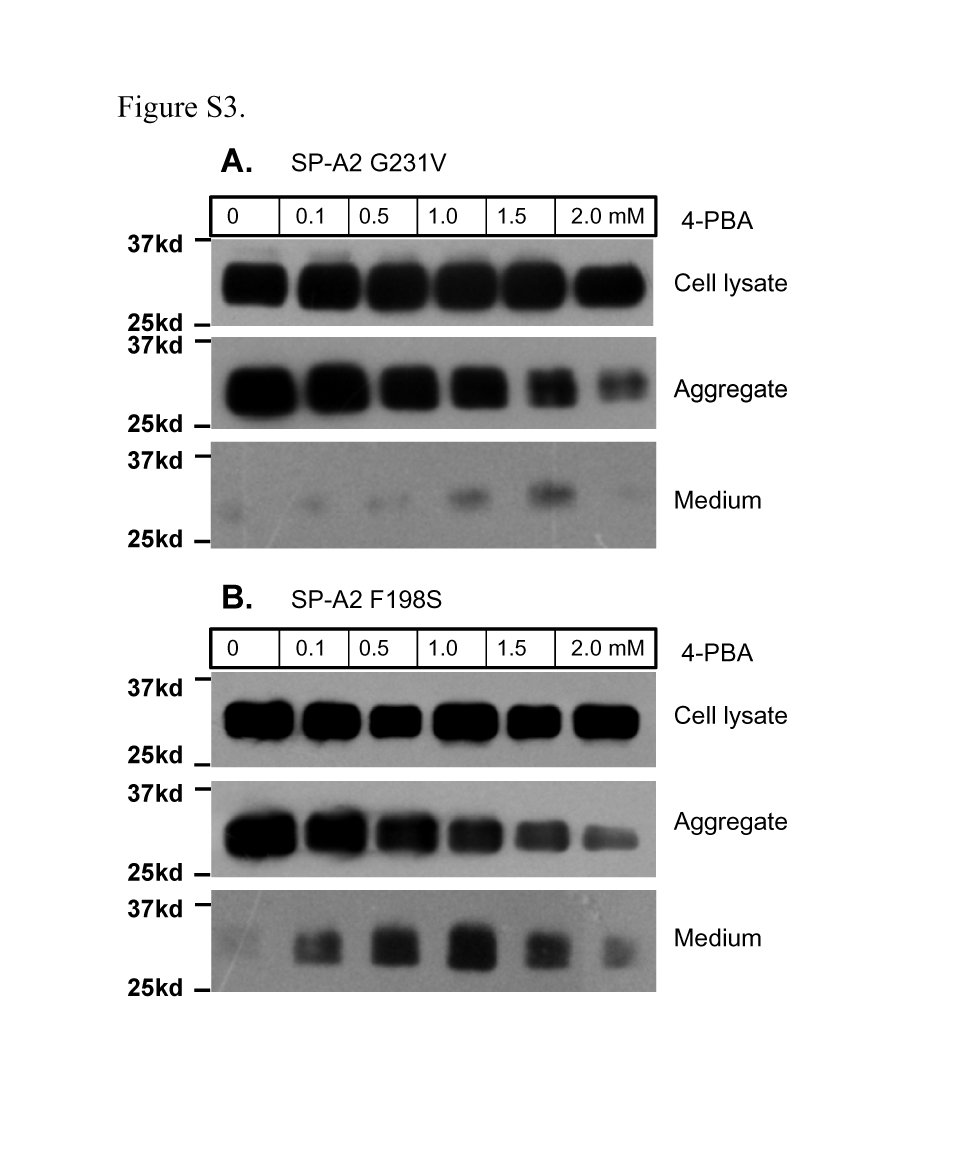

Supplement: Figure S3 — 4-PBA does-dependent decreases NP-40-insoluble aggregates of G231V and F198S variants in CHO-K1 cells. Transient expressing SP-A2 wild-type and variants CHO-K1 cells were incubated with different concentrations of 4-PBA for 48 h and the NP-40-insoluble and -soluble from cell lysate and medium were collected and analyzed by SDS-PAGE and immunoblotting. (TIF) [file pone.0046559.s003.tif]
