# Supplementary material for: Human Surfactant Protein A2 Gene Mutations Impair Dimmer/Trimer Assembly Leading to Deficiency in Protein Sialylation and Secretion
Source: PLoS One. 2012 Oct 3;7(10):e46559. doi: 10.1371/journal.pone.0046559 (PMC3463533; doi:10.1371/journal.pone.0046559)
Supplement: Table S1 — Primer sequences used in plasmid construction, mutagenesis and RT-PCR. (DOC) [file pone.0046559.s004.doc]

**Table. S1. P**rimers for SP-A2 constructs and RT-PCR

| Primers name | Primer sequence |
| --- | --- |
| SP-A2 –F (cloning) | 5’-GAA TTC GTC GAC ATG TGG CTG TGC CCT CTG GCC-3’ |
| SP-A2 -R (cloning) | 5’-GAA TTC ACT AGT TCA GAA ATC ACA GAT GGT CAG TC-3’ |
| SP-A2 G231V-F | 5’-GTG GAG ATG TAC ACA GAT GTG CAG TGG AAT GAC AGG AAC-3’ |
| SP-A2 G231V-R | 5’-GTT CCT GTC ATT CCA CTG CAC ATC TGT GTA CAT CTC CAC-3’ |
| SP-A2 F198V-F | 5’-CCC AGC CCT GGA GAC TCC CGC TAC TCA GAT GGG -3’ |
| SP-A2 F198V-R | 5’-CCC ATC TGA GTA GCG GGA GTC TCC AGG GCT GGG-3’ |
| SP-A2 Q223K-F | 5’-GCA GGT CGG GGA AAA GAG AAG TGT GTG GAG ATG TAC ACA-3’ |
| SP-A2 Q223K-R | 5’-TGT GTA CAT CTC CAC ACA CTT CTC TTT TCC CCG ACC TGC-3’ |
| SP-A2 N207S-F | 5’-TCA GAT GGG ACC CCT GTA AGC TAC ACC AAC TGG TAC CGA-3’ |
| SP-A2 N207S-R | 5’-TCG GTA CCA GTT GGT GTA GCT TAC AGG GGT CCC ATC TGA-3’ |
| SP-A2 N207T-F | 5’-TCA GAT GGG ACC CCT GTA ACC TAC ACC AAC TGG TAC CGA-3’ |
| SP-A2 N207T-R | 5’-TCG GTA CCA GTT GGT GTA GGT TAC AGG GGT CCC ATC TGA-3’ |
| SP-A2 N207A-F | 5’-CAG ACG GGA CCC CTG TAG CCT ACA CCA ACT GGT ACC-3’ |
| SP-A2 N207A-R | 5’-GGT ACC AGT TGG TGT AGG CTA CAG GGG TCC CGT CTG-3’ |
| SP-A2 N207Q-F | 5’-CAG ACG GGA CCC CTG TAC AGT ACA CCA ACT GGT ACC-3’ |
| SP-A2 N207Q-R | 5’-GGT ACC AGT TGG TGT ACT GTA CAG GGG TCC CGT CTG-3’ |
| SPA2 d(100-133)-1 | 5’-GAA GAC CTT CTC TCC TAC TGG AAG CCC TGG AGG GCC -3’ |
| SPA2 d(100-133)-2 | 5’- GGC CCT CCA GGG CTT CCA GTA GGA GAG AAG GTC TTC-3’ |
| SPA2 d(100-133)-3 | 5’- GTG TGC TGG AAT TCG TCG ACA TGT GGC TGT GCC CTC TG-3’ |
| SPA2 d(100-133)-4 | 5’-TAT CTG CAG AAT TCT CAG AAC TCA CAG ATG GTC AGT CG -3’ |
| SP-A2 –F (RT-PCR) | 5’-CCT CTG GCC CTC ACC CTC ATC TT-3’ |
| SP-A2 –R (RT-PCR) | 5’-GCG GCC GCC TGC TCT GG-3’ |
| GAPDH-F | 5’-ACC ACA GTC CAT GCC ATC AC-3’ |
| GAPDH-R | 5’-TCC ACC ACC CTG TTG CTG TA-3’ |
